# Mayer–Rokitansky–Kuster–Hauser Syndrome: From Radiological Diagnosis to Further Challenges—Review and Update

**DOI:** 10.3390/diagnostics16010138

**Published:** 2026-01-01

**Authors:** Calin Schiau, Csaba Csutak, Anca Ileana Ciurea, Roxana Pintican, Ioana-Teofana Dulgheriu, Simona Manole

**Affiliations:** 1 Department of Radiology and Medical Imaging, Faculty of Medicine, “Iuliu Hatieganu” University of Medicine and Pharmacy, 400012 Cluj-Napoca, Romania; 2 Department of Radiology, Emergency Clinical County Hospital Cluj-Napoca, 400006 Cluj-Napoca, Romania; 3 Department of Radiology, “Prof. Dr. Ion Chiricuta” Institute of Oncology, 400015 Cluj-Napoca, Romania; 4 Department of Radiology, “Niculae Stancioiu” Heart Institute, 400001 Cluj-Napoca, Romania

**Keywords:** MRKH, tip 2, uterine agenesis, vaginal agenesis, neovagina, Müllerian agenesis, ectopic kidney, pelvic MRI, amenorrhea, Müllerian abnormalities, infertility, uterine transplantation

## Abstract

Mayer–Rokitansky–Küster–Hauser (MRKH) syndrome encompasses a range of Müllerian duct anomalies characterized by congenital absence of the uterus and the upper two-thirds of the vagina in young women who otherwise exhibit normal endocrine function and a 46,XX karyotype. MRKH syndrome can occur in an isolated form (type I) or in association with other congenital anomalies (type II or MURCS association), which may include renal, vertebral, auditory, and cardiac defects. It represents one of the most frequent causes of primary amenorrhea, affecting approximately 1 in every 4000–5000 women. MRKH syndrome often remains undiagnosed until a patient presents with primary amenorrhea, despite normal development of secondary sexual characteristics. Both genetic and non-genetic factors have been proposed as contributing to abnormal embryonic development, although the exact etiopathogenesis remains unclear. Imaging plays a key role in the evaluation of genital tract anomalies, allowing non-invasive and comprehensive assessment. Alongside physical examination and pelvic ultrasound, pelvic MRI is essential for identifying the presence of rudimentary uterine tissue. MRKH syndrome can have profound and lasting psychological impacts, making it essential for patients and their families to receive counseling both before and throughout treatment. A range of therapeutic options—both surgical and non-surgical—have been proposed for managing MRKH syndrome. Vaginal dilation remains the first-line treatment, as it offers high success rates with minimal risk of complications. Vaginoplasty is considered a second-line option for patients who do not respond to dilation therapy. Additionally, uterine transplantation and gestational surrogacy provide opportunities for women with MRKH syndrome to achieve biological motherhood. This review provides an updated overview of Mayer–Rokitansky–Küster–Hauser (MRKH) syndrome, encompassing its etiological, clinical, diagnostic, psychological, therapeutic, and reproductive aspects. We also present a case involving a 19-year-old woman with MRKH syndrome who presented with primary amenorrhea, highlighting the crucial role and advantages of MRI in diagnosis, differential assessment, and treatment planning.

## 1. Introduction

### 1.1. Background

Mayer–Rokitansky–Küster–Hauser (MRKH) syndrome encompasses a spectrum of Müllerian duct anomalies, characterized by congenital absence of the uterus and the upper two-thirds of the vagina in individuals with a normal 46,XX karyotype and normal endocrine function.

The condition was first described in 1829 by Mayer, who reported partial and complete vaginal duplications in four stillborn infants, often accompanied by other anomalies such as cleft lip, limb malformations, and cardiac or urological defects. Rokitansky later expanded on this work in 1838, documenting 19 adult autopsy cases of uterovaginal agenesis, three of which included unilateral renal agenesis. In 1961, Hauser and Schreiner emphasized the importance of distinguishing MRKH syndrome from testicular feminization (now known as androgen insensitivity syndrome), as both involve underdeveloped vaginal structures but differ significantly in etiology and management [1].

In this review, we explore the most commonly recommended diagnostic approaches and management strategies, and summarize available data regarding outcomes related to sexual function, psychosocial adjustment, and reproductive options in patients with MRKH syndrome. We also present the case of a 19-year-old woman, with primary amenorrhea diagnosed with MRKH syndrome type II, highlighting the critical role of MR imaging in the diagnostic workup.

Over the past decade, notable progress has been made in MRKH research, particularly in the fields of genetics, non-surgical treatment, and fertility restoration through uterus transplantation. This review provides a comprehensive overview of imaging diagnostics, therapeutic options, psychological implications, and evolving reproductive possibilities for individuals with MRKH syndrome.

The psychosexual impact of the condition should not be underestimated. Clinical care must prioritize comprehensive counseling and a supportive, empathetic dialogue with the patient. Management of vaginal agenesis typically begins with non-invasive vaginal dilation therapy following thorough counseling and education. Surgical options are available for patients who do not respond to or decline dilation therapy.

A groundbreaking development in fertility treatment for MRKH syndrome was the first successful clinical trial of uterus transplantation (UTx), conducted by a Swedish team and culminating in the first live birth in Gothenburg in 2014. This landmark achievement demonstrated the feasibility of biological motherhood in women with MRKH syndrome and has since inspired successful UTx procedures in multiple countries worldwide [2].

### 1.2. Objectives

The main objectives of this review are to summarize current knowledge on the epidemiology, embryological basis, and clinical presentation of MRKH syndrome, to evaluate available diagnostic approaches and their limitations, to compare contemporary management options, including surgical and non-surgical pathways. At the end, we will discuss psychosocial considerations, and areas where further investigation is needed to improve patient care and long-term outcomes.

## 2. Methods

This narrative review presents an update of Mayer–Rokitansky–Küster–Hauser (MRKH) syndrome on its etiologic, clinical, diagnostic, psychological, therapeutic and reproductive aspects. We also report a case of MRKH syndrome, type II (syndromic form), in a 19-year-old woman who presented with primary amenorrhea, stressing the role and benefit of MRI in the differential diagnosis.

To identify the related articles, a comprehensive search was conducted via PubMed, Scopus, Embase, Cochrane Library, and Google Scholar from 2010 to 2025. The keywords used during the search included, but were not limited to “amenorrhea”, “MRKH”, “Müllerian abnormalities”, “uterine agenesis”, “vaginal agenesis”, “neovagina”, “pelvic MRI”, “infertility”, “uterine transplantation”.

We included original research articles, clinical trials, observational studies, and case series involving human subjects, published in English, with special focus on the pathology of Mayer–Rokitansky–Küster–Hauser (MRKH) syndrome. All other articles were excluded. The initial search identified 234 articles. Following removal of duplicates and application of inclusion criteria, 62 articles were retained for analysis. Additional relevant literature was identified through backward citation tracking. Given the methodological variability across the available literature, a narrative synthesis approach was adopted.

## 3. Definition and Diagnosis Criteria

MRKH syndrome is a congenital disorder characterized by the absence of the uterus and complete absence or the upper two-thirds of the vagina, in individuals with a normal 46,XX karyotype and fully developed secondary sexual characteristics. It is classified as a Class I Müllerian duct anomaly [3].

MRKH represents a rare form of complete agenesis of the paramesonephric (Müllerian) duct derivatives—namely, the uterus, cervix, and upper vagina—in otherwise phenotypically and genotypically normal females with intact endocrine function [4].

Affected individuals present with normal external genitalia and typically undergo spontaneous puberty, exhibiting normal thelarche and pubarche.

The clinical hallmark of MRKH syndrome is primary amenorrhea, often leading to diagnosis during adolescence. MRKH accounts for approximately 16% of primary amenorrhea cases, making it the second most common cause after ovarian insufficiency [5].

**Figure 1:**
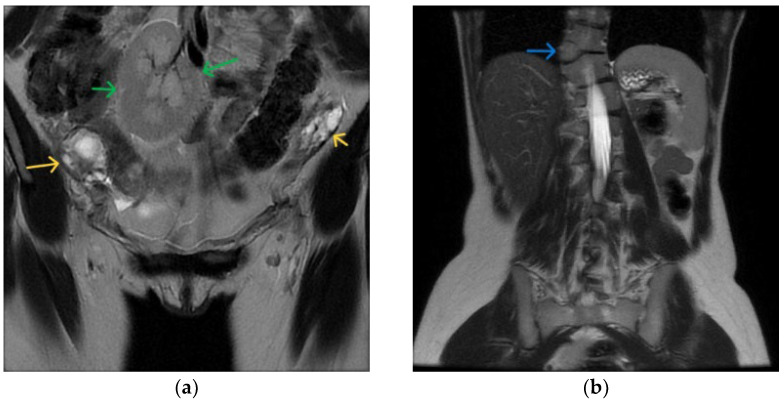

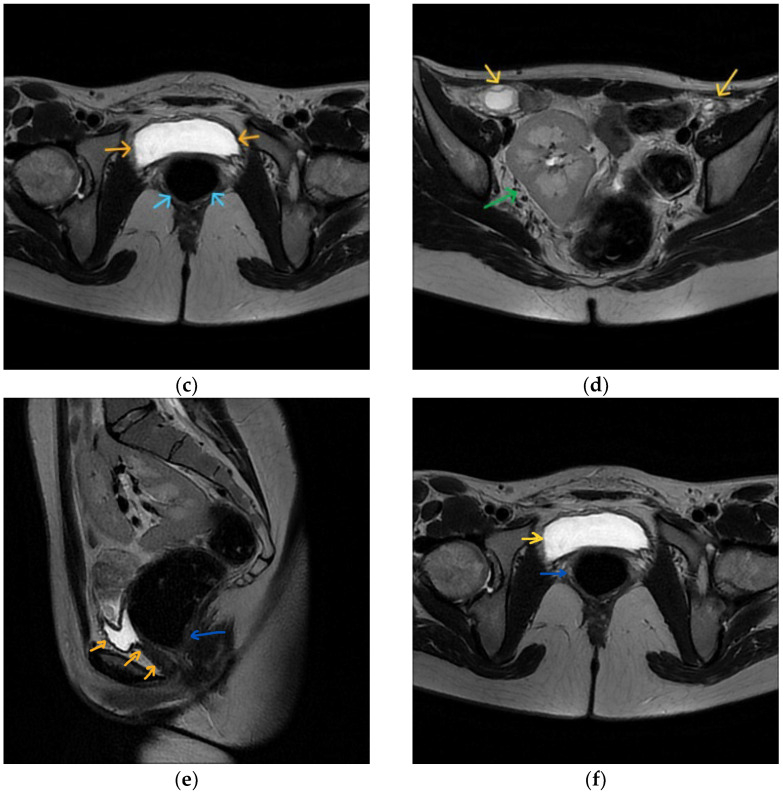
MRI images of the patient, with a series of objectified anomalies, all compatible with MRKH syndrome type 2. Coronal (**a**,**b**), axial (**c**,**d**,**f**) and sagittal (**e**) views. Bladder (orange arrows), rectum (light blue arrows), ovaries (yellow arrows), ectopic kidney (green arrows), hemivertebra (dark blue arrows) are outlined.

MRKH syndrome can be classified into two subtypes:

- Type I (isolated form): limited to uterovaginal agenesis.
- Type II (syndromic form): associated with extragenital anomalies, particularly involving the renal, skeletal, and cardiovascular systems (Figure 1).

Among those with MRKH type II, a subset of patients may present with MURCS association—a more severe phenotype involving Müllerian agenesis, renal anomalies, and cervicothoracic spine abnormalities—representing the most complex clinical form of the syndrome [3].

## 4. Clinical Presentation and Diagnostic Work-Up in MRKH Syndrome

### 4.1. Clinical Presentation

#### 4.1.1. Clinical Onset

The first clinical sign is typically primary amenorrhea, defined as the absence of menstruation by age 16 despite normal puberty and secondary sexual development.

Patients usually present with a normal female phenotype, a 46,XX karyotype [1], and normal, functional ovaries without evidence of androgen excess [6].

#### 4.1.2. Associated Symptoms

Dyspareunia or apareunia.

Cyclic pelvic or abdominal pain, caused by accumulation of hemorrhagic material in uterine buds with a functioning endometrium; in the post-pubertal period.

#### 4.1.3. Physical Examination

Completed puberty with normal secondary sexual characteristics (Tanner stage 5 pubic hair and breast development).

Normal external genitalia, but the vagina is reduced to a 2–7 cm vaginal dimple [1].

Examination should be performed by a pediatric/adolescent gynecologist or a disorder in sex development (DSD) specialist, with consideration of the patient’s age and motivation. Vaginal examination should be avoided in prepubertal adolescents.

### 4.2. Diagnostic Work-Up

#### 4.2.1. Imaging

Ultrasonography (US): transperineal or transabdominal; reveals absence of the uterus and presence of ovaries.

Magnetic Resonance Imaging (MRI): gold standard for diagnosing uterovaginal agenesis. It is superior to CT for visualizing Müllerian structures and endometrial tissue in uterine remnants. Also identifies ovaries and extragenital malformations. High inter-rater agreement with laparoscopy [7].

Laparoscopy: rarely used for diagnosis; indicated when symptomatic uterine remnants require removal [7].

Renal imaging (US/MRI): recommended to exclude renal malformations, present in 30% of cases [5].

Additional screening: skeletal, ear, and cardiac anomalies should be evaluated when relevant clinical findings are present [5]: EOS scan, otorhinopharyngeal assessment and echocardiography.

#### 4.2.2. Genetic Testing

Chromosomal microarray analysis can identify copy number variations, with a positivity rate of 16–20%, but clinical interpretation remains uncertain [8].

Not required for diagnosis.

#### 4.2.3. Laboratory Tests

FSH, LH, and estradiol levels are usually normal.

Androgen levels are generally normal; however, recent data suggest biochemical (subclinical) hyperandrogenemia in 50% of patients, pending further validation [6].

## 5. MRKH Syndrome Classification—Ovarian and Associated Anomalies

### 5.1. Ovarian Findings

In MRKH syndrome, the ovaries are usually present and functional. They are often positioned more cranially than normal and typically lie lateral to the external iliac arteries, which is likely due to absent Fallopian tubes. Ovarian anomalies are uncommon, occurring in approximately 5–10% of cases, and may include unilateral agenesis, ectopic ovaries, polycystic ovaries, streak ovaries, or, rarely, ovarian tumors [1].

### 5.2. MRKH Syndrome Subtypes

Type I, also called the isolated form or “Rokitansky sequence,” involves isolated uterovaginal aplasia.

Type II, known as the MURCS association, features uterovaginal aplasia along with other malformations. Kidney anomalies are present in around 40% of Type II cases, with 15% showing unilateral renal agenesis. Hearing impairment occurs in approximately 10% of patients, and skeletal abnormalities are observed in 10–12% [1,9].

### 5.3. Associated Malformations

#### 5.3.1. Skeletal Abnormalities

Skeletal abnormalities occur in 30–40% of MURCS cases, predominantly affecting the spine. Spinal malformations include scoliosis (about 20%), asymmetric, fused, or wedged vertebrae, Klippel–Feil syndrome (cervical vertebral fusion with short neck, low hairline, and restricted neck motion), Sprengel’s deformity, rib agenesis or malformations, and spina bifida [9].

Facial and limb anomalies can also be present, such as brachymesophalangy, ectrodactyly, duplicated thumb, absent radius, atriodigital dysplasia (Holt–Oram-like), and facial asymmetry [10].

#### 5.3.2. Hearing Impairment

Hearing impairment is reported in 10–25% of MURCS patients [11]. Most cases involve conductive hearing loss caused by middle ear malformations, such as stapedial ankylosis. Sensorineural defects of varying severity may also occur. Some patients present with auditory canal dysplasia or malformed external ears [10].

#### 5.3.3. Cardiac Malformations

Cardiac anomalies are less common but, when present, tend to be severe and often resemble Holt–Oram or velocardiofacial syndromes. Reported defects include aortopulmonary window, atrial septal defect [12], and conotruncal defects such as pulmonary valve stenosis or Tetralogy of Fallot [13].

## 6. Embryology

The urinary and genital systems develop in close association during embryogenesis. In humans, the female reproductive tract consists of the fallopian tubes (oviducts), uterus, cervix, and vagina. The fallopian tubes, uterus, cervix, and upper two-thirds of the vagina originate from the paramesonephric (Müllerian) ducts (PMDs), whereas the lower third of the vagina develops from the urogenital sinus. MRKH syndrome arises from complete agenesis or aplasia of the PMDs, resulting in the absence of the uterus and the upper portion of the vagina.

In individuals with MRKH syndrome, this developmental defect leads to a range of abnormalities, most frequently vaginal atresia and absence of the uterus and cervix. Since the lower third of the vagina arises from ectodermal cells, it may present as a short, blind-ending pouch or dimple in the perineum, while the external genitalia generally appear normal. Ovarian development and function are typically unaffected, as the ovaries originate from the primitive mesoderm and migrate to the pelvis during fetal development [4].

This anomaly is thought to arise during embryogenesis, resulting from arrested development of the paramesonephric (Müllerian) ducts around the seventh week post-fertilization. Between the eighth and twelfth weeks of gestation, the Müllerian ducts give rise to the fallopian tubes, uterus, cervix, and upper three-quarters of the vagina [14]. Disruptions in development during this critical period can lead to agenesis of these structures. Concurrently, the kidneys, ureters, and bladder develop between approximately the sixth and twelfth weeks of gestation. In many cases, residual structures, such as a blind-ending vaginal pouch or a rudimentary uterus, may be observed [1,3].

## 7. Epidemiology and Pathology

MRKH syndrome has a reported incidence of approximately 1 in 4000–5000 live female births [3].

Numerous studies in Europe and Asia have evaluated patients with both type I and type II MRKH syndrome. Among the largest European cohorts are those by Oppelt et al. (2006), Strubbe et al., Rall et al., and Weijenborg et al. [1,11,15,16]. In Asia, significant cohorts were studied by Deng et al. (2019) and Pan, H.-X. et al. (2019), with a primary focus on the prevalence of extragenital anomalies [17,18]. The following were observed:

Renal malformations are the most common extragenital abnormalities in MRKH syndrome, observed in 30–40% of European cohorts. Unilateral renal agenesis (URA) is the most frequent anomaly, accounting for roughly half of all renal malformations associated with MRKH syndrome. Notably, URA is often accompanied by complete absence of the ipsilateral Müllerian duct, indicating a close developmental link between the kidneys and Müllerian ducts [19]. Other renal anomalies may include pelvic kidney, duplex kidney, and horseshoe kidney. In contrast, studies in Chinese cohorts report a lower prevalence of renal malformations, with Deng et al. documenting 13% and Pan et al. reporting 5%, suggesting potential inter-ethnic phenotypic differences in MRKH syndrome [8,17].

Skeletal anomalies are the second most common extragenital manifestation, affecting approximately 10–40% of patients depending on the extent of evaluation. These anomalies predominantly involve the axial skeleton, including scoliosis, Klippel–Feil anomaly, hemivertebrae, and rib aplasia, with less frequent involvement of the extremities. Cardiac abnormalities are reported in fewer than 5% of patients, examples being pulmonary valve stenosis and atrial septal defects [1]. Hearing impairments, including both sensorineural and conductive types such as external auditory meatus atresia or stapedial ankylosis, are generally reported in less than 5% of cases, though they are not routinely assessed [15].

In a small subset of patients, MRKH syndrome presents with a severe phenotype resembling VACTERL association, characterized by vertebral defects, anal atresia, cardiac anomalies, tracheoesophageal fistula or esophageal atresia, renal defects, and limb abnormalities. Even fewer patients present with isolated anorectal malformations [20].

## 8. Etiology and Genetics

### 8.1. Etiology

The etiology of MRKH syndrome remains largely unclear; however, the spectrum of malformations observed suggests a developmental field defect involving organ systems closely related during embryogenesis [21]. More specifically, MRKH syndrome may originate from an initial disruption of the intermediate mesoderm, which by the end of the fourth week of fetal development affects the blastema of the cervicothoracic somites and the pronephric ducts. These structures subsequently guide the differentiation of the mesonephroi and, later, the Wolffian and Müllerian ducts [21].

Most cases of MRKH syndrome are sporadic [22]. Nevertheless, familial cases have been reported, with occurrences of MRKH and other extragenital anomalies following an autosomal dominant inheritance pattern with incomplete penetrance. This suggests a genetic component in at least a subset of cases [3,22]. Despite the documentation of familial cases, the genetic basis remains incompletely understood because many molecular studies have not included analyses of blood relatives of the probands, limiting the ability to identify de novo mutations or assess inheritance patterns [23].

Human tissue patterning and organ morphogenesis result from complex interactions between genetic factors, soluble morphogens, chemical cues, and mechanical forces [22]. Given this complexity, multiple etiologies have been proposed for MRKH syndrome, including monogenic, oligogenic, polygenic, multifactorial, environmental factors, regulatory mechanisms, and somatic genetic events during development [3].

### 8.2. Genetics

#### 8.2.1. Latest Genetic Findings in MRKH Syndrome

Recent genetic investigations have highlighted the heterogeneity and complexity underlying MRKH syndrome, illustrating that no single gene or pathway accounts for most cases. Large reviews emphasize recurrent involvement of genes located in the 17q12 region—particularly HNF1B and LHX1—though their penetrance and phenotypic expressivity remain variable [8,24]. Functional genomics work by Thomson et al. provided some of the strongest mechanistic evidence to date, showing that loss of HNF1B function disrupts Müllerian duct development and produces uterine and renal anomalies consistent with MRKH type II [25]. Broader sequencing efforts have identified additional candidate genes, including WNT4, TBX6, GLI3, DLG5, and others, but the majority of these findings remain limited to isolated cases or small cohorts without reproducible genotype–phenotype correlation. Large-scale phenotyping by Pietzsch et al. similarly supports the interpretation that MRKH is genetically diverse, with many variants classified as of uncertain significance [26]. Overall, while emerging studies continue to expand the list of plausible developmental genes, the lack of consistent replication and the absence of robust functional validation mean that the genetic basis of MRKH remains only partially understood, and current evidence supports a multifactorial etiology that may include germline, somatic, and epigenetic contributions.

#### 8.2.2. Genetic Aspects Related to the Presented Case—Type II MRKH Syndrome

Among candidate genes, GREB1L (growth regulation by estrogen in breast cancer 1-like) has emerged as particularly significant. GREB1L is an androgen-regulated gene and a co-activator of the retinoic acid receptor (RAR) gene, with expression levels influencing renal system cellular differentiation, morphogenesis, and homeostasis in vertebrates [15,22]. In 2019, Herlin et al. conducted whole-exome sequencing (WES) in a three-generation family in which two female cousins had type II MRKH syndrome with unilateral renal agenesis, and two male relatives had renal agenesis. The study identified a co-segregating missense variant in GREB1L [3].

Subsequent studies have supported this association. Jacquinet et al. reported four multiplex families with either type II MRKH syndrome or uterovaginal aplasia in fetuses, along with renal malformations, in which pathogenic GREB1L variants were detected via WES [27]. Mutations in GREB1L have also been found in cases of isolated bilateral renal agenesis and deafness, both of which are extragenital anomalies seen in type II MRKH [26]. Although GREB1L mutations have not yet been linked to cardiac malformations, there is evidence of its involvement in ventricular development [10,15].

Jolly et al. reported 18 GREB1L variants in 16 unrelated individuals from a cohort of 590 MRKH probands and in two individuals from a congenital scoliosis cohort. This study concluded that GREB1L, a retinoic acid–responsive gene expressed in the intermediate mesoderm, is critical for the development of the uterus and kidneys and is associated with a spectrum of human disorders, including isolated uterine agenesis. Using quantitative phenotypic analyses in a worldwide multiethnic cohort, the study strengthened the association of GREB1L with both isolated MRKH type I and syndromic MRKH type II [23].

GREB1L now appears to be the first gene showing a strong association with type II MRKH syndrome accompanied by kidney anomalies, following an autosomal dominant inheritance pattern with incomplete penetrance [10]. These findings underscore the importance of obtaining a detailed family history and considering radiological examinations to detect subtle genitourinary anomalies in relatives of affected patients [23].

## 9. Diagnostic Methods and Radiographic Features (Except for MRI Examination)

Once MRKH syndrome is diagnosed, a comprehensive evaluation is essential to identify associated malformations. Because renal and skeletal anomalies are often asymptomatic, at a minimum, transabdominal ultrasonography and spinal radiography should be performed. If there is suspicion of hearing impairment or cardiac anomalies, further assessments, including audiogram and echocardiography, are recommended [1].

Additionally, obtaining a detailed family history is important, and depending on the findings, screening of the patient’s relatives may be warranted [6,27].

### 9.1. Transabdominal Ultrasonography

Ultrasound is a simple, noninvasive first-line tool for evaluating suspected Müllerian aplasia. It typically demonstrates the absence of uterine structures between the bladder and rectum. However, a quadrangular retro vesical structure may be misinterpreted as a hypoplastic or juvenile uterus; this corresponds to the vestigial lamina located beneath the peritoneal fold, where the uterosacral ligaments attach. Unlike normal uterine tissue, the vestigial lamina contains no cavity and lacks the hyperechogenic line representing the endometrial lining [1,28].

Ultrasonography also allows assessment of renal anomalies and ovarian presence. Its accuracy, however, is operator-dependent, results may vary across examinations, and it does not provide the neutrality required for surgical planning [1].

### 9.2. Hysterosalpingography

Although this technique is well-established for evaluating uterine Müllerian anomalies, it is not useful in MRKH syndrome due to the hypoplasia or agenesis of the uterus and the upper two-thirds of the vagina.

### 9.3. Celioscopy

Celioscopy is an invasive procedure that requires hospitalization and anesthesia. It is generally reserved for cases where the diagnosis remains uncertain after ultrasonography and/or MRI [3].

This technique is mainly indicated in women who are likely to undergo interventional treatment, such as the construction of a neovagina. It allows for precise assessment of the anatomical location and abnormalities of the uterus, the presence of tubal remnants, the vestigial lamina, and the ovaries [3].

### 9.4. Biological Status

As part of the diagnostic evaluation, assessment of sex hormones is considered essential, since ovarian function is usually preserved and hormonal levels remain within the normal range. Chromosomal analysis must also be performed to distinguish MRKH syndrome from other conditions presenting with primary amenorrhea, making it an indispensable tool, as diagnosis is based on exclusion [1].

Patients with MRKH syndrome consistently have a normal 46,XX karyotype without visible chromosomal abnormalities. Endocrine parameters—including plasma levels of follicle-stimulating hormone (FSH), luteinizing hormone (LH), and 17β-estradiol—are within normal limits, confirming normal ovarian function. Likewise, there are no external or endocrine signs of hyperandrogenism, which is supported by normal plasma levels of testosterone, delta-4-androstenedione, 17-hydroxyprogesterone, and dehydroepiandrosterone [28].

## 10. MRI Evaluation Role in MRKH Syndrome: Technique, Image Analysis, Clinical Interpretation and Surgical Implications

Magnetic resonance imaging (MRI) is the cornerstone of detailed anatomical assessment in MRKH syndrome, providing high-resolution, multiplanar visualization that complements clinical examination and informs individualized patient management. While ultrasound is often used as a first-line screening tool, it has limited sensitivity for small uterine remnants, subtle endometrial tissue, and associated anomalies; MRI, particularly high-resolution T2-weighted sequences in axial, sagittal, and coronal planes, offers superior soft-tissue contrast and spatial resolution [24].

### 10.1. Technique and Image Analysis

Uterine agenesis and hypoplasia are best assessed on sagittal images, while transverse images provide optimal evaluation of vaginal agenesis [1]. Adapted protocols, including thin-slice imaging, 3D reconstructions, and occasionally contrast enhancement, enhance the detection of small or hypoplastic uterine buds and aid in differentiating MRKH subtypes [24]. These features allow accurate MRKH classification: Type I (isolated uterovaginal agenesis) versus Type II (syndromic MRKH with associated renal, skeletal, or auditory anomalies).

### 10.2. Clinical Interpretation and Surgical Implications

Precise imaging is critical for surgical planning, particularly for neovagina creation or reconstructive procedures. MRI informs decisions about the optimal surgical approach, available tissue planes, and vascular relationships, minimizing intraoperative risk and improving functional outcomes. Moreover, MRI findings guide multidisciplinary management, such as nephrology consultation for renal anomalies or orthopedic evaluation for vertebral malformations (see Table 1), and are essential for patient counseling regarding reproductive options and surgical expectations [9].

Clinicians interpreting MRI results should systematically evaluate:

- Presence, size, and morphology of uterine remnants;
- Patency and length of the vaginal canal;
- Renal and urinary tract anomalies;
- Skeletal and soft-tissue abnormalities;
- Relationship of pelvic structures, especially bladder and rectum.

Integration with clinical guidelines: MRI should always be interpreted alongside clinical assessment. Detection of renal anomalies, for instance, mandates early nephrology involvement. Functional uterine remnants may influence counseling about fertility preservation or reconstructive options. MRI thus serves as both a diagnostic and planning tool, bridging anatomical characterization with patient-centered surgical and multidisciplinary care [8].

A key clinical feature in MRKH syndrome is cyclic pelvic pain during puberty, often caused by hemorrhagic accumulation within uterine buds containing functioning endometrium [29]. Before MRI, this could only be suspected clinically or confirmed via laparoscopy with intraoperative ultrasonography or pathological examination. MRI now allows noninvasive identification of functioning endometrial tissue by visualizing cavitated uterine buds with a characteristic target pattern. Detection of such buds guides surgical excision to prevent complications [3].

Furthermore, identifying well-developed ovaries may support the potential creation of a small functional uterus, and locating a vaginal bud between the bladder and rectum can guide vaginal reconstruction [30]. MRI’s accuracy permits postponing invasive laparoscopic exploration until the patient is ready for surgery, while also providing information on structures that may not be visible during laparoscopy [9].

In conclusion, MRI is an indispensable tool for the diagnosis and anatomical evaluation of MRKH syndrome, offering detailed pelvic assessment and aiding surgical planning [19]. Close collaboration between radiologists and the surgical team is critical, as MRI findings, including urinary anomalies, directly influence surgical strategies such as the Vecchietti procedure [9]. Accurate differentiation of MRKH syndrome from other Müllerian anomalies is crucial not only for guiding surgical management but also for addressing reproductive potential and psychological considerations [31].

## 11. Differential Diagnosis

Müllerian aplasia should be considered in patients presenting with primary amenorrhea and normal secondary sexual characteristics. The initial step in differential diagnosis is the exclusion of gonadal dysgenesis. Key conditions to consider include congenital absence of the uterus and vagina (aplasia or agenesis), isolated vaginal atresia, androgen insensitivity syndrome (AIS), and WNT4 gene defects (see Table 2) [10].

### 11.1. Androgen Insensitivity Syndrome (AIS)

AIS, formerly termed testicular feminization, is an X-linked recessive disorder caused by mutations in the androgen receptor (AR) gene, resulting in incomplete or absent masculinization of the external genitalia in chromosomally male (46,XY) individuals. AIS is classified into two forms.

#### 11.1.1. Complete Androgen Insensitivity Syndrome (CAIS, Morris Syndrome)

CAIS patients have a 46,XY karyotype with female external genitalia, a blind-ending vagina, absent uterus, and normal breast development with sparse pubic hair. Some cases of 17-hydroxylase/17,20-lyase deficiency due to biallelic CYP17A1 mutations can produce a similar phenotype, with normal or ambiguous external genitalia, absent uterus, and shortened vagina [1,10]. Chromosomal analysis is essential to differentiate MRKH syndrome from 46,XY disorders of sex development (DSDs).

#### 11.1.2. Partial Androgen Insensitivity Syndrome (PAIS)

PAIS presents with a spectrum ranging from mildly virilized female external genitalia (e.g., clitorimegaly) to undervirilized male genitalia (e.g., hypospadias or reduced penile size) [1,6]. Affected individuals have normal testes with normal testosterone production and conversion to dihydrotestosterone (DHT), distinguishing PAIS from 5-alpha reductase deficiency. Because the testes secrete normal Müllerian-inhibiting factor (MIF), these patients lack fallopian tubes, uterus, and proximal vagina [10].

### 11.2. WNT4 Defects

Loss-of-function mutations in the WNT4 gene cause developmental abnormalities of sexual differentiation. Clinically, WNT4 defects may mimic MRKH syndrome, but evidence of hyperandrogenism in an otherwise phenotypically female patient should prompt suspicion of WNT4 involvement [1,6].

### 11.3. Isolated Vaginal Atresia

Vaginal atresia is observed in several syndromes, notably Winter syndrome (renal, genital, and middle ear anomalies, OMIM #267400) and McKusick–Kaufman syndrome (hydrometrocolpos, postaxial polydactyly, congenital heart malformations, OMIM #236700), caused by mutations in MKKS on chromosome 20p12 [3,10]. Clinically, patients present with pelvic pain and cryptomenorrhea. Unlike MRKH syndrome, vaginal atresia does not confer irreversible sterility, as surgical correction can allow for pregnancy [10].

### 11.4. Müllerian Derivative Aplasia

Some patients exhibit Müllerian aplasia alongside abnormal karyotypes involving the X chromosome, including mosaicisms (45,X/46,XX; 46,XX/45,X0), structural rearrangements (46,X,del(X)(pter-q22); 46,X,i(Xq)), or complex karyotypes [10].

### 11.5. Turner Syndrome

Turner syndrome is the most common genetic cause of pubertal delay and primary amenorrhea. Diagnosis is confirmed by karyotype analysis (45,X) and is associated with elevated FSH levels. Clinical features include short stature, webbed neck, low posterior hairline, shield chest, and delayed puberty due to ovarian insufficiency [32].

### 11.6. CYP17A1 Deficiency (17α-Hydroxylase Deficiency)

Mutations in CYP17A1 impair the synthesis of sex steroids and cortisol. Affected individuals present with hypertension and hypokalemia. Females retain the uterus and vagina, whereas 46,XY individuals develop female external genitalia, a blind-ending vagina, and intra-abdominal testes [32].

Laboratory findings typically reveal low sex steroid levels, elevated gonadotropins, elevated progesterone, and mineralocorticoid-induced hypertension, providing a pathognomonic profile [32,33].

## 12. Treatment

Differentiating MRKH syndrome from other Müllerian anomalies, particularly malformations of the distal genital tract, is critical not only because surgical strategies differ but also because reproductive potential and psychological impact vary among conditions.

Young women diagnosed with MRKH often experience profound anxiety and psychological distress upon learning they lack a uterus and upper vagina. Comprehensive counseling for both the patient and family, ideally beginning at diagnosis and continuing throughout treatment, is strongly recommended [7]. Participation in group programs and patient associations can provide additional support [16]. Psychological adjustment and a supportive medical approach are key determinants of future decisions regarding neovagina creation and management of infertility [34].

It is essential to discuss all treatment options, emphasizing that creating a functional vagina is a gradual process. Surgical procedures require postoperative maintenance through regular intercourse or vaginal dilation to ensure long-term success. Since 2002, the American College of Obstetricians and Gynecologists (ACOG) has recommended dilation therapy as the first-line treatment due to its high overall success rate (90–96%), minimal invasiveness, low complication rates, and low cost [7]. Supervision by a healthcare professional experienced in dilation therapy is crucial to maximize compliance and outcomes. Callens et al. support this approach and suggest laparoscopic Vecchietti vaginoplasty as the preferred second-line surgical option when dilation therapy fails [35].

Regardless of the selected approach, treatment should be preceded by counseling regarding expected outcomes and potential complications, ensuring that the patient is fully mature and motivated. For some patients, opting not to undergo treatment may also be a valid choice.

### 12.1. Non-Surgical and Surgical Procedures

Treatment for MRKH can be divided into two main approaches: creation of a new vaginal cavity or replacement using a pre-existing canal lined with mucosa, such as a bowel segment. Neovagina creation should be offered only when the patient is emotionally mature and ready to initiate sexual activity [35,36].

#### 12.1.1. Non-Surgical Creation of a Neovagina

The most widely used non-surgical method is the Frank dilation technique. Vaginal dilation represents the recommended first-line, non-surgical intervention for neovaginal creation in patients with MRKH syndrome, with reported success rates exceeding 90% when performed with adequate frequency and technique [8,24].

This non-invasive approach requires thorough patient counseling and stepwise instruction. Vaginal dilators (Hegar candles) are applied first by the clinician and subsequently by the patient, gradually increasing in size and diameter. Daily application for at least 20 min is recommended, with treatment duration ranging from six weeks to several months. Success rates range from 78% to 92%, with rare complications such as urethritis, cystitis, vesico- or rectovaginal fistula, or secondary prolapse. This method is limited to patients with a sufficiently deep vaginal dimple (2–4 cm) [35,36,37,38,39].

Despite its efficacy, adherence remains a significant clinical challenge. Patients often report discomfort, perineal tension, or difficulties integrating regular dilation sessions into daily routines, which may lead to suboptimal compliance and prolonged treatment duration [8,9].

Psychological factors—including anxiety, limited sexual self-efficacy, and distress related to the diagnosis—also influence adherence and can negatively affect outcomes if unaddressed [24,26]. Evidence suggests that structured counseling, early education, and multidisciplinary follow-up improve adherence by facilitating realistic expectations and supporting emotional adjustment [8,24].

Long-term outcomes are generally favorable, with most individuals achieving adequate vaginal length, satisfactory sexual function, and sustained anatomical results; however, some patients may require periodic maintenance dilation to prevent regression [9,24].

Overall, the effectiveness of dilation therapy is contingent not only on mechanical technique but also on continuous psychosocial support and individualized patient-centered care.

#### 12.1.2. Surgical Creation of a Neovagina

Surgical approaches vary, with technique selection based on surgeon experience and patient-specific factors (see Table 3). Major techniques include:

- Abbe-McIndoe Operation: involves creating a space between the rectum and bladder, inserting a mold covered with a skin graft, and performing postoperative vaginal dilation. Modifications include spontaneous epithelialization or using peritoneum, labial grafts, or synthetic materials [40].
- Vecchietti Procedure: combines surgical and non-surgical elements. A traction device, often placed laparoscopically, gradually dilates the vaginal dimple with a plastic “olive” attached subperitoneally, producing reliable outcomes [41].
- Sigmoidal Colpoplasty (Baldwin Vaginoplasty): uses a 12–18 cm segment of sigmoid colon to create a neovagina. While highly effective, prolonged postoperative care may be required for optimal sexual function. This method is generally avoided in patients undergoing uterine transplantation due to risks of adhesions and contamination from bowel mucosa [42,43].
- Davydov Procedure: involves mobilizing peritoneum to form a neovagina, which undergoes spontaneous squamous epithelialization over approximately six months. The procedure is minimally invasive, leaves no visible scars, and avoids graft-related complications [44].
- Williams Vulvovaginoplasty: uses labia majora flaps to form a short neovagina (about 3 cm). Limitations include risk of contraction and the need for ongoing dilation or sexual activity to maintain patency.
- Tissue-Engineered Techniques: recent advances utilize autologous vulvar tissue or tissue-engineered biomaterials to construct a neovagina [36,38].
- Acellular Graft Surgery: acellular grafts, such as intestinal submucosa or other extracellular matrix scaffolds, are increasingly used in neovagina construction. These grafts provide a biocompatible framework that supports host tissue ingrowth and epithelialization without introducing donor cells, reducing the risk of immune rejection. Over time, the scaffold is gradually remodeled into functional vaginal tissue with a normal mucosal lining, which is advantageous for maintaining vaginal microbiota and potentially for uterine transplantation procedures [36,38]. Key advantages include being non-crosslinked (which promotes cell colonization) and avoiding the risks associated with synthetic mesh or the need for autologous tissue donors [38].

Methods based on dilation of the vaginal dimple provide a neovagina with normal mucosal lining, which is advantageous in the context of uterine transplantation by supporting a normal vaginal microbiota and enabling accurate cervical biopsy assessment [36,38].

### 12.2. Advantages and Limitations

Callens et al. reviewed outcomes, benefits, and drawbacks of various procedures [35]. Surgical methods are limited by invasiveness, anesthesia requirement, graft-specific complications, and the need for continued postoperative dilation [3,35]. Dilation therapy carries risks of poor compliance, prolonged treatment duration, discomfort, and rare urethral dilation [3,35].

### 12.3. Key Points in Treatment

- Psychological preparation is essential prior to any intervention.
- Non-surgical creation of a neovagina should be the first-line therapy.
- Surgery is reserved for patients in whom dilation therapy fails, and postoperative dilation remains necessary.
- Infertility remains one of the most challenging aspects for patient acceptance.

## 13. Infertility and Uterus Transplantation (UTx)

### 13.1. Infertility

Women with MRKH syndrome are classified among females with absolute uterine factor infertility (AUFI), which includes those with either a complete absence of the uterus or a non-functional uterus incapable of supporting embryo implantation and pregnancy. Traditionally, the primary option for achieving motherhood in women with MRKH syndrome and other forms of AUFI has been legal adoption [45].

Currently, gestational surrogacy (GS) represents an alternative pathway to achieve genetic motherhood, and following adoption by the birth-giving mother, also legal motherhood. In this approach, in vitro fertilization is performed using oocytes from the MRKH woman and sperm from her partner, and the resulting embryo is transferred into the uterus of a gestational surrogate. Surrogacy arrangements may be commercial or altruistic, often involving a close relative such as a mother or sister as the carrier, depending on the legal framework of the specific country or state [46]. However, gestational surrogacy is prohibited in the Nordic countries and in many other regions worldwide due to ethical, religious, or legal considerations, or a combination thereof [45].

Uterus transplantation (UTx) has recently emerged as the first definitive infertility treatment for women with MRKH syndrome, offering the possibility of full motherhood—including gestational, genetic, and legal aspects—from the outset.

### 13.2. Uterus Transplantation (UTx) Surgery

#### 13.2.1. Procedure

UTx is generally performed through a sub-umbilical midline incision. The vaginal vault is carefully dissected free from the bladder and rectum, facilitated by cleavage of the uterine remnant along the midline. The external iliac arteries and veins are then dissected and cleared of surrounding tissue to allow for vascular anastomosis. The chilled and perfused uterus is transferred from the back table into the pelvis. End-to-side anastomoses are performed between the uterine vessels of the graft and the recipient’s external iliac vessels. Following reperfusion, the vagina is opened for end-to-end vaginal anastomosis, after which the uterus is secured to the surrounding ligaments [47].

Minimal invasive surgery, including robotic-assisted laparoscopy, is expected to become the main surgical approach for live donor procedures in the near future and, subsequently, for recipient surgeries [48]. Advantages of this approach include reduced tissue trauma and a shortened hospital stay of 1–2 days.

#### 13.2.2. Immunosuppression

Patients undergoing UTx receive standard induction therapy similar to that used in kidney transplantation. Maintenance therapy primarily involves the calcineurin inhibitor tacrolimus [49]. A key feature of UTx is that it is a temporary transplantation; immunosuppressive therapy is required only for the duration the graft is in place and can be discontinued once the uterus is removed—typically within five years, after the recipient has delivered the desired number of children. This approach minimizes the long-term side effects of calcineurin inhibitors, such as nephrotoxicity, which is particularly important for MRKH patients, some of whom have a solitary kidney [49].

#### 13.2.3. Efficacy

Although still in the early stages of human application, UTx has already proven to be an effective fertility treatment, with a take-home baby rate exceeding 80% among patients with graft survival beyond six months [49]. The efficiency of UTx is expected to improve further with ongoing advances in medical techniques.

#### 13.2.4. Factors Influencing Success

The age of both donor and recipient, as well as whether the donor is living or deceased, are important determinants of transplant outcomes. Many studies suggest that ideal recipients are under 35–40 years of age, while donors may be older, including postmenopausal women, with an upper age limit of 55–65 years [2,50].

#### 13.2.5. Supporting Literature

Brännström et al., 2015 [50], demonstrated that UTx is feasible using live donors, even postmenopausal, as a treatment for MRKH-related infertility.

Briget da Graca et al., 2021, reported long-term uterine viability and function after live-donor transplantation, noting that asymptomatic rejection episodes could be managed with temporary glucocorticoid therapy [51].

Castellón et al., 2017, highlighted UTx as an effective treatment for infertility due to congenital or acquired uterine absence, particularly in MRKH patients [52].

Zaami et al., 2017, concluded that UTx can restore reproductive capability and improve quality of life, though it is a complex procedure with significant health risks [53].

Chmel et al., 2018, found that nearly two-thirds of women with surgically created neovaginas were interested in undergoing UTx and motivated to pursue the procedure [54].

## 14. Psychological Symptoms and Impact of the Treatment

### 14.1. Emotional Response

Studies included in this review indicate that receiving a diagnosis of MRKH syndrome is a highly stressful experience, often eliciting negative emotional reactions, heightened sensitivity to perceived differences, and a diminished sense of femininity, particularly due to infertility [36,55]. This underscores the importance of comprehensive care and psychological counseling.

MRKH patients are frequently adolescents, a developmental stage marked by emotional and physical vulnerability, which necessitates heightened awareness and sensitivity from healthcare providers regarding patients’ emotions, reactions, and coping mechanisms [7,55]. Many patients are accompanied by their parents during consultations, who themselves experience emotional distress. Psychological counseling can support parents—especially mothers—in providing effective emotional support to their daughters [56].

Women with MRKH often exhibit symptoms of anxiety, depression, psychoticism, eating disorders, and low self-esteem. However, studies indicate that, following appropriate treatment, overall health-related quality of life (HRQoL) can be favorable [36].

Three primary sources of psychological vulnerability have been identified in women with MRKH syndrome:

- Concerns about genital image and discomfort during sexual activity, including anxieties about genital function.
- Worries that a partner might detect their condition, although research suggests that most men cannot distinguish a neovagina from a natural one [36].
- The quality of the mother–daughter relationship, with some women perceiving maternal involvement as excessive or intrusive during treatment [55,56].

### 14.2. Sexual Function

Most studies have assessed sexual function and well-being using quantitative tools such as the Female Sexual Function Index (FSFI) and the Female Sexual Distress Scale-Revised (FSDS-R), particularly to evaluate outcomes following neovaginal therapy [57]. The creation of a neovagina, whether by surgical or non-surgical methods, can reduce concerns about genital difference and improve sexual well-being, especially when managed by a multidisciplinary team [58]. However, isolated treatment is often insufficient to fully address the multiple sources of distress experienced by women with MRKH syndrome. Psychological counseling should therefore be routinely offered, including assessment of beliefs about genital appearance and partner perceptions, as well as guidance on disclosure to intimate partners [16].

HadaviBavili et al. reported that all surgical techniques for neovagina creation positively impacted sexual function. Nonetheless, total FSFI scores remained significantly lower than those of healthy women. Domain-specific analysis revealed that lubrication (*p* < 0.05) and satisfaction (*p* < 0.05) were particularly affected in patients undergoing vaginoplasty [28].

### 14.3. Family Dynamics

Reproductive options should be clearly explained, emphasizing that these women typically have functional ovaries. Although they cannot carry a pregnancy due to uterine absence or malformation, a variety of alternatives exist for achieving parenthood.

The World Health Organization (WHO) recommends a holistic, biopsychosocial approach to sexuality, emphasizing self-esteem, sexual relations, and the development of coping strategies to navigate the psychosexual challenges associated with MRKH syndrome [16].

Overall, MRKH syndrome is complex, and its management requires a multidisciplinary approach. Care teams should include healthcare professionals for ongoing counseling and support, experts in genital malformations or disorders of sexual development, and sexologists or psychologists.

### 14.4. Ethical and Psychosocial Considerations in Advanced Reproductive Options for MRKH Syndrome

Ethical discussions surrounding advanced reproductive interventions for individuals with MRKH syndrome—particularly gestational surrogacy and uterine transplantation (UTx)—remain insufficiently developed in the literature. Although these technologies have expanded reproductive possibilities, access to them differs greatly across healthcare systems, legal frameworks, and institutional policies, which can indirectly shape patients’ reproductive autonomy without being fully acknowledged in clinical reports [7,59,60].

In parallel, several analyses of UTx programs and experimental reproductive procedures report incomplete or limited discussion of alternative options, psychosocial implications, or long-term medical commitments associated with immunosuppression and repeated surgical interventions [7,51].

Beyond procedural considerations, the treatment of psychosocial and reproductive-ethical issues in MRKH-related literature is often superficial, despite consistent evidence that patients face substantial emotional, relational, and identity-related challenges associated with congenital uterine absence, infertility, and decisions regarding dilation, neovaginoplasty, or reproductive assistance [61,62]. Many publications refer only briefly to psychological support or counseling, without detailing structured assessments, long-term follow-up, or the integration of mental-health specialists into care pathways. In addition, discussions of treatment risks—particularly in the context of UTx—are frequently imbalanced. Some studies highlight technical feasibility or successful pregnancies while giving limited attention to graft failure, complications from immunosuppression, donor morbidity, repeated surgical procedures, or the physical and emotional burdens placed on recipients and donors alike [51,59,63].

Taken together, these gaps emphasize the need for more rigorous ethical reporting, standardized consent practices, and comprehensive psychosocial frameworks to ensure that reproductive decision-making in MRKH syndrome is informed, balanced, and patient-centered.

## 15. Teaching Point and Conclusions

Mayer–Rokitansky–Küster–Hauser (MRKH) syndrome is one of the most frequent causes of primary amenorrhea. Magnetic Resonance Imaging (MRI) plays a crucial role in visualizing the vaginal, cervical, and uterine morphology, which is essential for guiding treatment planning and patient management.

While ultrasound is valuable for assessing the genitourinary tract—particularly for detecting associated renal anomalies—MRI offers greater anatomical detail and accuracy. It is also less invasive and more cost-effective than laparoscopy, making it a preferred imaging modality in the majority of cases.

Despite the psychological impact of this condition, modern surgical techniques can correct anatomical anomalies, allowing for normal sexual function. Furthermore, reproductive potential can often be achieved through assisted reproductive technologies. As such, accurate diagnosis and comprehensive management of MRKH syndrome are vital for optimizing both physical and emotional outcomes for affected individuals.

## 16. Future Directions in MRKH Syndrome Research and Management

Advancements in the management of MRKH syndrome continue to evolve, and several key areas warrant further investigation. Uterine transplantation has emerged as a promising option for select patients wishing to pursue gestational motherhood. Although still experimental in many countries, ongoing research is needed to refine surgical techniques, reduce immunosuppression risks, and expand access and long-term outcome data.

Psychological support remains a critical component of care, as MRKH syndrome can significantly impact emotional well-being, identity, and interpersonal relationships. Future studies should aim to develop standardized, multidisciplinary psychological interventions tailored to different stages of diagnosis and treatment.

Additionally, progress in genetic research may help to unravel the underlying etiologies of MRKH syndrome, which remain largely unclear. Identifying specific genetic mutations or epigenetic factors could improve early diagnosis, enable genetic counseling, and potentially guide future therapeutic strategies.

An integrated approach combining medical, surgical, psychological, and genetic research is essential to enhance quality of life and reproductive autonomy for individuals affected by MRKH syndrome.

## Figures and Tables

**Table 1:** The relationship between MRI Evaluation–Clinical Interpretation–Surgical Implications in MRKH syndrome.

MRKH Subtype	MRI Findings	Clinical Significance	Surgical Implications
Type I (isolated)	Bilateral absence or hypoplastic uterine buds; normal renal anatomy	Confirms isolated uterovaginal agenesis	Standard neovagina creation; no systemic anomaly evaluation needed
Type II (syndromic)	Uterine buds absent or hypoplastic; renal anomalies (agenesis, ectopia, duplication); vertebral anomalies; occasionally auditory defects	Identifies patients requiring multidisciplinary assessment	Surgical planning must account for renal position, skeletal anomalies, and tissue availability; tailored approach for neovagina or uterine reconstructive procedures
Hypoplastic uterus with functional endometrium	Small uterine remnants with endometrial signal on T2	May inform future fertility options (e.g., uterine transplant, assisted reproduction)	Surgical caution to preserve functional tissue; possible hormonal evaluation

**Table 2:** Summary of MRKH syndrome differential diagnosis [10].

Condition	Reproductive Anatomy	Secondary Traits	Karyotype	Lab Pattern	Menstruation	Malignancy Risk
MRKH/MURCS	Absent vagina, Absent uterus; normal ovaries	Normal breasts and pubic hair	46,XX	High testosterone	Primary amenorrhea	No increased risk
ISOLATED VAGINAL ATRESIA	Variable vagina; uterus present; normal ovaries	Normal secondary development	46,XX	Normal labs	Primary amenorrhea	No increased risk
WNT4 SYNDROME	Absent uterus; masculinized ovaries	Hyperandrogenism; Normal breasts and pubic hair.	46,XX	High testosterone	Primary amenorrhea	Indirect; monitoring required
AIS	Absent uterus; testes present	Normal breasts; sparse pubic hair	46,XY	Normal/High FSH and LH	Primary amenorrhea	High risk
TURNER SYNDROME	Normal uterus; streak ovaries	Delayed puberty	45,X	High FSH	Primary amenorrhea	Elevated if Y present
CYP17A1 DEFICIENCY	Underdeveloped uterus and genitalia	Underdeveloped traits	46,XX/XY	High FSH; high progesterone; low estrogen	Irregular	Low, in 46,XY

**Table 3:** Comparison Table—Surgical Options for MRKH syndrome.

Technique	Typical Indications	Reported Success (Anatomic/Functional)	Major Risks/Complications
Laparoscopic Vecchietti (including modified/traction variants)	Patients preferring minimally invasive, single-operation neovagina after failed/declined dilation	95–98% anatomic/functional success in large series (high patient satisfaction).	Intraoperative visceral/vascular injury (rare), postoperative pain, short hospital stay; requires traction device and short postop dilation.
Davydov (laparoscopic peritoneal) vaginoplasty	Patients wanting autologous mucosal lining without graft; also used in revisions	High anatomic/functional success comparable to Vecchietti in many reports.	Urinary/rectal injury (rare), introital stenosis, need for temporary dilation; potential for adhesion formation.
McIndoe (skin graft) vaginoplasty	Patients in whom other methods are not available/surgeon familiarity; still used in many centers	Good functional results historically; variable long-term satisfaction due to graft issues.	Graft failure, donor-site scarring, prolonged mold/dressing care, need for secondary procedures.
Sigmoid (intestinal) vaginoplasty	Revision cases, failed prior procedures, need for longer self-lubricating neovagina	Durable length and lubrication; success often high but variable.	Higher surgical complexity: anastomotic leak, ileus, mucosal prolapse, introital stenosis, infection; reoperation rates reported.
Modified techniques (SIS grafts, tissue-engineered grafts, flap variations)	When desire to avoid donor skin or for scar-minimizing approaches; experimental/recently adopted	Early series report satisfactory outcomes comparable to established techniques in small cohorts.	Technique-specific risks (graft integration, immune reaction for some materials), limited long-term data.

## Data Availability

No new data were created or analyzed in this study. Data sharing is not applicable to this article.
